# A Robust Monocular and Binocular Visual Ranging Fusion Method Based on an Adaptive UKF

**DOI:** 10.3390/s24134178

**Published:** 2024-06-27

**Authors:** Jiake Wang, Yong Guan, Zhenjia Kang, Pengzhan Chen

**Affiliations:** 1 School of Intelligent Manufacturing, Taizhou University, Taizhou 318000, China; jinshanhekk@163.com (J.W.); guanyong@ecjtu.edu.cn (Y.G.); shrunk_cybm@163.com (Z.K.); 2 School of Electrical and Automation Engineering, East China Jiaotong University, Nanchang 330013, China

**Keywords:** visual ranging, monocular vision, binocular vision, adaptive Unscented Kalman Filter (UKF), robust estimation, depth propagation

## Abstract

Visual ranging technology holds great promise in various fields such as unmanned driving and robot navigation. However, complex dynamic environments pose significant challenges to its accuracy and robustness. Existing monocular visual ranging methods are susceptible to scale uncertainty, while binocular visual ranging is sensitive to changes in lighting and texture. To overcome the limitations of single visual ranging, this paper proposes a fusion method for monocular and binocular visual ranging based on an adaptive Unscented Kalman Filter (AUKF). The proposed method first utilizes a monocular camera to estimate the initial distance based on the pixel size, and then employs the triangulation principle with a binocular camera to obtain accurate depth. Building upon this foundation, a probabilistic fusion framework is constructed to dynamically fuse monocular and binocular ranging using the AUKF. The AUKF employs nonlinear recursive filtering to estimate the optimal distance and its uncertainty, and introduces an adaptive noise-adjustment mechanism to dynamically update the observation noise based on fusion residuals, thus suppressing outlier interference. Additionally, an adaptive fusion strategy based on depth hypothesis propagation is designed to autonomously adjust the noise prior of the AUKF by combining current environmental features and historical measurement information, further enhancing the algorithm’s adaptability to complex scenes. To validate the effectiveness of the proposed method, comprehensive evaluations were conducted on large-scale public datasets such as KITTI and complex scene data collected in real-world scenarios. The quantitative results demonstrate that the fusion method significantly improves the overall accuracy and stability of visual ranging, reducing the average relative error within an 8 m range by 43.1% and 40.9% compared to monocular and binocular ranging, respectively. Compared to traditional methods, the proposed method significantly enhances ranging accuracy and exhibits stronger robustness against factors such as lighting changes and dynamic targets. The sensitivity analysis further confirmed the effectiveness of the AUKF framework and adaptive noise strategy. In summary, the proposed fusion method effectively combines the advantages of monocular and binocular vision, significantly expanding the application range of visual ranging technology in intelligent driving, robotics, and other fields while ensuring accuracy, robustness, and real-time performance.

## 1. Introduction

With the rapid development of autonomous driving technology, the requirements for the environmental perception capabilities of intelligent vehicles are increasingly high. As a core module of the perception system, accurate, real-time, and robust target ranging has become crucial. Currently, the perception systems of most autonomous vehicles rely on higher cost sensors, such as LiDAR, radar, and the high-precision Global Positioning System (GPS). However, cameras, with their lower cost, provide richer perceptual information, making them a more attractive alternative [1]. However, factors such as complex lighting conditions, dynamic traffic participants, and texture-poor scenes pose serious challenges to visual ranging technology. According to the statistics from large-scale autonomous driving datasets like KITTI, the average error in monocular visual ranging can reach up to 20.3%, while the inaccuracy rate of binocular visual ranging is also as high as 12.5%. This indicates that, on average, for every five kilometers driven, one kilometer of ranging results is unreliable, which poses a significant challenge to the vehicle’s safe decision-making and path planning. Therefore, achieving high-precision and high-robustness visual ranging in complex environments has become a key issue that the field of autonomous driving urgently needs to address.

Visual ranging technology mainly consists of two categories: monocular visual ranging and binocular visual ranging. Monocular ranging, as a typical visual positioning system, has been widely applied in various fields.

Monocular distance measurement estimates the distance from the camera to the target by analyzing the scale changes of the target in a sequence of images captured by a single camera. Early work was mostly based on target detection and size priors, such as the visual localization method based on a single-frame image proposed by Chen [2], which uses the height of a vehicle’s undercarriage to estimate the distance to the front vehicle or the head-to-shoulder width ratio of pedestrians to estimate the distance to pedestrians. The limitation of these methods is that they heavily rely on prior knowledge, and their performance drops significantly when the target is occluded or partially visible. In recent years, the academic community has begun to introduce deep learning into monocular distance measurement. For example, the method proposed by Pereira [3] based on using Gauss–Newton nonlinear regression to minimize projection residuals has been extended to monocular distance measurement, and Huang [4] proposed a scale estimation network to estimate the size of the detected object. However, existing monocular visual distance measurement methods generally face the problem of distance drift. The main reason is that monocular imaging lacks absolute scale information, making it difficult to eliminate the cumulative error in the measurement results.

Binocular vision distance measurement restores the depth of the target through the disparity information from two cameras, allowing for the acquisition of the absolute distance. Traditional binocular distance measurement includes steps such as rectification, matching, and post-processing [5]. Representative work includes an improved Census-transform-based stereo matching algorithm proposed by Guo [6], and a stereo-matching method based on an attention-based hybrid cost volume computation module proposed by Dai [7]. However, the drawbacks of traditional methods are their high computational load and insufficient robustness, especially being prone to mismatches in scenarios with weak textures and repetitive textures. To improve the accuracy and speed of matching, some scholars have begun to explore the use of deep learning for binocular matching. For example, Feng [8] mentioned the use of MobileNet to enhance the speed of feature extraction. However, existing binocular distance-measurement methods still face issues such as low disparity resolution and the tendency for small targets to be lost, with performance being less than ideal in long-distance and complex scenarios.

In summary, monocular vision distance measurement and binocular vision distance measurement each have their strengths and weaknesses. The advantage of monocular distance measurement lies in its low cost and flexible deployment, but it is prone to scale drift; the advantage of binocular distance measurement is that it can obtain the absolute distance, but it is greatly affected by lighting and texture changes. To leverage the complementary nature of both methods, some scholars have proposed fusion frameworks. For example, Lu [9] mentioned the use of binocular vision to assist in eliminating the processing errors between the actual model and the theoretical mathematical model of monocular positioning, and Peng [10] proposed a self-supervised monocular depth-estimation network that learns through binocular geometric correlation. These works indicate that the fusion of visual distance measurement is an effective way to address complex environmental perception problems. However, most existing works adopt simple weighted averaging or posterior optimization strategies, lacking modeling of the confidence level of different distance measurement information, which makes it difficult to effectively suppress outliers. In addition, the adaptability of existing fusion methods is insufficient, lacking the ability to dynamically adjust the fusion strategy according to environmental characteristics, and the accuracy and robustness need to be improved in complex scenarios such as open roads.

In response to the aforementioned issues, this paper proposes a novel fusion method for monocular and binocular ranging. We establish a probabilistic fusion framework based on the Unscented Kalman Filter (UKF), which explicitly models the ranging errors of monocular and binocular vision as Gaussian distributions. Additionally, we introduce an adaptive observation-noise-adjustment mechanism to achieve the dynamic weighting of ranging confidence. Building upon this foundation, we further design an adaptive prior-generation strategy based on deep hypothesis propagation. This strategy autonomously adjusts the noise priors during the UKF prediction phase according to the environmental characteristics, enhancing the algorithm’s adaptability to dynamic scenes. The main contributions of this paper are as follows:

1. A novel probabilistic fusion framework is proposed, which unifies the ranging results of monocular and binocular vision along with their uncertainties under the Unscented Kalman Filter (UKF) framework, achieving dynamic recursive optimization of the ranging errors. Compared with deterministic fusion methods, probabilistic fusion can better model the confidence levels of different information sources, thereby enhancing the accuracy and robustness of the fusion process.
2. An adaptive observation-noise-adjustment mechanism is introduced, which dynamically updates the UKF’s observation covariance matrix based on the current fusion residual, mitigating the impact of outliers and extreme values. Compared with fixed-weight fusion strategies, adaptive adjustment allows for the real-time allocation of the weights according to the quality of the ranging information, enhancing the adaptability of the fusion algorithm to interfering factors.
3. A strategy for adaptive prior generation based on deep hypothesis propagation was designed, which utilizes neural networks to predict depth hypotheses and their uncertainties from monocular image sequences, and dynamically adjusts the UKF’s state transition noise accordingly. This enables the fusion algorithm to intelligently adjust the noise priors during the prediction phase according to the scene characteristics, further enhancing its adaptability to open environments.
4. Extensive evaluations were conducted on the large-scale public KITTI dataset and complex scene datasets collected in real-world conditions. Through multi-faceted experiments including quantitative metrics, ablation studies, and qualitative analysis, the accuracy, robustness, real-time performance, and superiority of the proposed fusion method were demonstrated, showcasing its application potential in the field of environmental perception for autonomous driving.

The research findings of this paper enrich and develop the technology of visual ranging. The proposed adaptive UKF fusion method provides a new solution for high-precision, high-robustness target distance perception in complex environments. Theoretically, the work in this paper is expected to promote the integration and innovation of probabilistic modeling and deep learning in the field of multi-sensor fusion. In terms of application, the method presented can significantly enhance the environmental perception performance of autonomous vehicles, providing reliable distance information for planning and control, thereby facilitating the development and implementation of autonomous driving technology.

## 2. Related Work

This section systematically reviews the visual ranging methods closely related to the research of this article, focusing on representative works of three types of methods: monocular ranging, binocular ranging, and fusion ranging. Their advantages and disadvantages are analyzed and discussed.

### 2.1. Monocular Visual Ranging

Monocular visual ranging utilizes the imaging principle of a monocular camera to estimate the distance from the camera to the target based on the geometric and appearance features of the target in the image. Based on whether prior knowledge of the target is required, it can be categorized into supervised ranging and unsupervised ranging.

Supervised distance measurement establishes a mapping relationship from images to distances by manually designing features or training deep models offline. Early research was mostly based on target detectors and real-size priors, such as estimating the distance to the front car using the height of a vehicle’s undercarriage or estimating the distance to pedestrians using the head-to-shoulder width ratio. The limitation of these methods is that they heavily rely on prior knowledge, and their performance drops significantly when the target is occluded or partially visible. To reduce dependence on priors, some scholars have proposed using deep learning to directly regress depth information from images. For example, Zheng [11] proposed a lightweight convolutional deep learning model that can extract object-specific distance information from monocular images. Liu [12] further introduced an attention mechanism into the depth estimation network, improving the regression accuracy for long-distance targets through global context modeling. Recently, lightweight CNNs such as MobileNet [8] and MonoDepth2 [13] have been used for monocular depth estimation, further enhancing the model’s speed and accuracy. However, supervised distance-measurement methods generally face challenges such as the scarcity of large-scale distance-labeled data and the high cost of manual annotation, and their generalization ability still needs to be further verified.

Unsupervised distance measurement aims to estimate camera motion and target distance using a sequence of monocular camera images, without relying on manual annotation. The mainstream approach combines monocular visual odometry with a depth estimation network, achieving joint optimization of network parameters and camera poses through optical flow constraints and motion consistency loss functions. For instance, Zhan [14] proposed a method that uses stereo sequences to learn depth and visual odometry. Another example is the novel unsupervised learning framework proposed by Liu [15], which learns forward and backward inertial sequences across multiple subspaces to produce motion features that are consistent in scale and independent of the environment, and selectively weights inertial and visual modalities to adapt to various scenarios and motion states. Although unsupervised methods are no longer constrained by manually annotated data, the learned depth predictions are still susceptible to interference from factors such as changes in lighting and moving objects, and their robustness in practical applications needs to be further improved.

The monocular ranging method employed in this paper is fundamentally similar to that of Jin [16]. It utilizes the pinhole perspective principle in establishing the monocular ranging model, defining the relationship between the camera coordinate system and the image coordinate system within the camera imaging model. A camera coordinate system, parallel to the X-Y plane and with the optical center as the origin, is established, with the ZC axis perpendicular to the X-Y plane. The camera’s focal length is used to convert image coordinates into actual spatial coordinates. Additionally, the YOLOv5 network is incorporated for object recognition. The center point of the object, once obtained, is then translated into a three-dimensional point in the world coordinate system through the ranging model, thereby calculating the real-world distance.

### 2.2. Binocular Visual Ranging

Binocular visual ranging utilizes images from two cameras with different perspectives to calculate the three-dimensional position of targets using triangulation principles, thereby obtaining distance information. It can be categorized into methods based on sparse feature points and methods based on dense pixels depending on the feature type.

The classic sparse binocular ranging method first extracts salient feature points from images, such as SIFT [17], ORB [18], etc., then establishes correspondences between left and right images through descriptor matching, and finally, utilizes epipolar geometry to solve for disparity and depth. However, the depth map obtained from sparse matching typically has low resolution, making it challenging to support high-precision target ranging. Therefore, pixel-based dense matching methods are gradually becoming the mainstream approach.

Traditional dense stereo matching algorithms, such as the stereo matching method, are based on an attention-based hybrid cost volume computation module proposed by Pantilie [19], which significantly improves the matching accuracy and efficiency. However, these methods have high computational complexity and are susceptible to interference from factors such as changes in lighting, weak textures, etc., as well as the improved Semi-Global Matching (I-SGM) algorithms mentioned by Guo [20]. In recent years, deep learning has been widely used in stereo matching and has made significant progress. For example, Huang [21] proposed the Pyramid Stereo Matching Network (PSMNet), which encodes and matches global contextual information through spatial pyramid pooling and 3D convolution, significantly surpassing traditional SGM methods on the KITTI stereo matching benchmark. Zhang [22] proposed a stereo matching algorithm based on the Minimum Spanning Tree (MST) cost aggregation, and Yang [23] proposed an improved stereo matching algorithm based on AANet. Liu [12] developed an effective enhanced YOLOX-tiny model by adding the Coordinate Attention (CA), CIoU, and Focus Loss to the original CNN, improving the mAP by 3.25Despite the continuous record-breaking performance in accuracy and speed by deep learning-based dense matching, existing CNN methods still face issues such as the loss of small targets and insufficient accuracy at long distances.

The binocular ranging method used in this paper is similar to the method described by Wei [24]. However, the binocular ranging method in this paper does not incorporate the improvements in the YOLOv5 and stereo matching parts as described in the literature. It simply combines the object-recognition capabilities of YOLOv5 with traditional binocular ranging to facilitate the subsequent fusion of range measurements for the same target.

### 2.3. Visual Ranging Fusion

Due to the respective advantages and disadvantages of monocular and binocular visual ranging—flexibility, but susceptibility to drift for the former, and higher accuracy, but constrained by the baseline for the latter—some scholars propose integrating both approaches to leverage their strengths and mitigate their weaknesses. Existing fusion strategies can generally be categorized into geometric-based deterministic methods and probability-based stochastic methods.

Deterministic fusion methods often use monocular distance measurement results as a prior constraint or post-processing condition for binocular matching, obtaining the final distance estimation through weighted averaging. For example, Jiang [25] uses monocular scale estimation to initialize the search range for binocular disparity, which results in positioning accuracy that is less affected by the drift of inertial components and prevents error accumulation. However, the limitation of such fusion methods is that they lack the consideration of the differences in confidence levels between monocular and binocular distance measurements, which can easily introduce significant errors in situations such as occlusions and sudden changes in lighting.

Stochastic fusion methods model the distance measurement from sensors as a probability distribution and estimate the posterior probability of the distance using Bayesian inference. For instance, Zeng [26] proposed a factor graph-based multi-sensor fusion framework that obtains the optimal distance estimation by optimizing the maximum a posteriori (MAP) estimate of the depth hypothesis. Although the aforementioned stochastic fusion takes into account the confidence levels of different observations to some extent, most assume that the observation noise from different information sources follows a fixed Gaussian distribution, lacking online adaptive modeling of the noise’s statistical properties. As a result, the accuracy and robustness in complex environments are still not optimal.

In recent years, some scholars have begun to explore the integration of deep learning into multi-sensor fusion. However, such deep learning-based fusion approaches are still immature, facing challenges such as high model complexity, low computational efficiency, and poor interpretability. Their reliability and safety in practical systems still require further validation.

**Figure 1:**
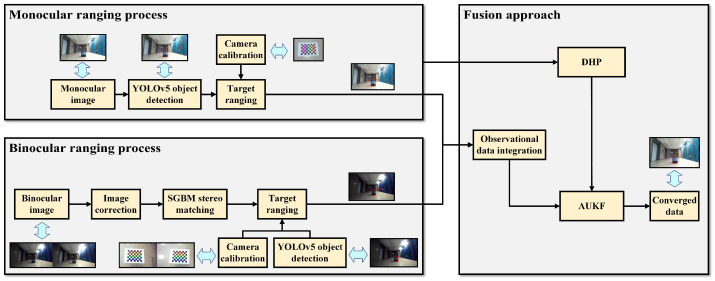
Overall system framework.

In summary, existing monocular and binocular ranging fusion methods still face three major challenges: (1) there is a lack of unified modeling of uncertainty for different visual information sources, making it difficult to accurately assess the quality of ranging and the differences in confidence levels; (2) the fusion process lacks a mechanism to adapt to environmental dynamics, and the accuracy and robustness in complex scenes need to be improved; (3) the utilization of image semantic information is not yet sufficient, and the guiding role of scene context clues on ranging has not been fully explored. In response to the above issues, this paper proposes a new framework for adaptive and robust monocular and binocular ranging fusion driven by a probabilistic graph. We first construct a factor graph model that represents the likelihood probabilities and uncertainties of monocular depth estimation and binocular disparity matching in a unified manner and obtain the optimal distance by inferring the maximum a posteriori (MAP) estimate of the depth hypothesis. On this basis, we design an adaptive Unscented Kalman Filter (UKF) scheme that dynamically adjusts the observation noise priors of different measurements according to the current fusion residual, in order to suppress the impact of outliers. Furthermore, we construct a depth hypothesis-propagation mechanism that uses deep learning to predict the depth prior and its confidence level from a sequence of monocular images, and guides the UKF filtering process in the autonomous generation of noise priors, thereby enhancing the adaptive ability of the fusion algorithm to complex dynamic scenes. The overall framework of this paper is shown in Figure 1. In this figure, “AUKF” refers to the adaptive Unscented Kalman Filter fusion framework and “DHP” stands for deep hypothesis propagation.

## 3. Monocular and Binocular Visual Ranging Fusion Method

This section elaborates in detail on the adaptive Unscented Kalman Filter (UKF) monocular and binocular visual ranging fusion method proposed in this paper. Firstly, we establish a unified state space representation based on a probabilistic graphical model, formalizing the fusion problem of monocular and binocular ranging into a Bayesian filtering problem. On this basis, we designed an adaptive UKF fusion framework that achieves the dynamic weighting of the ranging errors by introducing an observation noise self-calibration mechanism during the filtering process. Furthermore, to enhance the adaptability of the fusion algorithm to complex environmental changes, we also propose an adaptive-prior-generation strategy based on depth hypothesis propagation, which uses deep learning to predict the depth prior distribution and its uncertainty from a sequence of monocular images, guiding the state-prediction step of the UKF.

### 3.1. Problem Definition and Symbol Nomenclature

Consider an intelligent agent equipped with a monocular camera and a stereo camera. At discrete time step *k*, denote the camera pose as $T_{k}\in \mathrm{SE}\left(3\right)$ and the target to be measured as $O_{k}$. The monocular and stereo cameras provide distances $d_{k}^{mono}$ and $d_{k}^{stereo}$ for the target $O_{k}$, respectively. The objective of this paper is to design a fusion algorithm that dynamically weights $d_{k}^{mono}$ and $d_{k}^{stereo}$ to estimate the true distance $d_{k}^{gt}$ of $O_{k}$.

To describe the problem, we define the following symbols:

- $x_{k}={\left[d_{k},v_{k}\right]}^{T}$: the target state vector at *k*th moment, including the real distance $d_{k}$ and its rate of change $v_{k}$.
- $z_{k}^{mono},\;z_{k}^{stereo}$: the distance observations for monocular and binocular vision at the *k*th moment.
- $z_{k}={\left[z_{k}^{mono},z_{k}^{stereo}\right]}^{T}$: the fusion observation vector at the *k*th moment.
- $R_{k}^{mono},\;R_{k}^{stereo}$: the observed noise covariance of monocular vision and binocular vision at the *k*th moment.
- $R_{k}=diag\left(R_{k}^{mono},R_{k}^{stereo}\right)$: the converged observational noise covariance matrix at the *k*th moment.
- $Q_{k}$: the process noise covariance matrix at the *k*th moment.
- $P_{k}$: the state estimation error covariance matrix at the *k*th moment.
- ${\hat{x}}_{k}$: the estimated mean of the state at the *k*th moment.

### 3.2. Probabilistic Graphical Model

**Figure 2:**
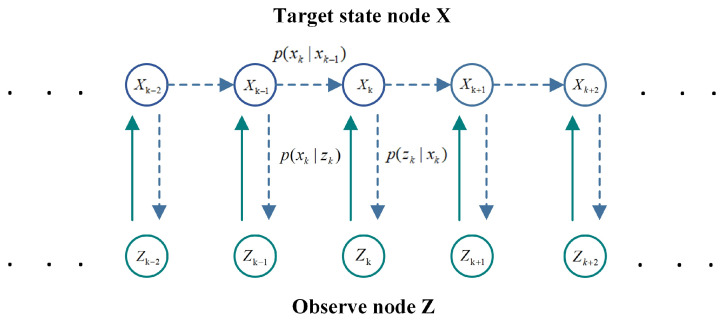
Probabilistic graphical model.

In order to uniformly represent the ranging results and uncertainty of monocular and binocular vision, we construct a probability graph model as shown in Figure 2. The model contains two kinds of nodes: target state node $X=x_{k}|k=1,\ldots ,K$ and observation node $Z=z_{k}|k=1,\ldots ,K$. The target state nodes represent the true distance to the target that is to be estimated and its rate of change, while the observation nodes represent the distance observations made by monocular vision and binocular vision at each moment.

In the figure, the solid lines represent the generative model and the dashed lines represent the inference model. To be specific, the generative model depicts the target state X and observational data Z cause-and-effect relationship between the state transition probability $p(x_{k}|x_{k-1})$ and observation probability $p(z_{k}|x_{k})$. The inference model reflects how to estimate the posterior distribution p(X|Z) of the target state X from the observed data z. The core of this fusion algorithm is to model the fusion problem of monocular distance measurement and binocular distance measurement as a temporal Bayesian inference problem. The hidden variable node $x_{k}$ in the figure represents the target state sequence, and the observation nodes $z_{k}^{mono}$ and $z_{k}^{stereo}$ represent the measured value sequence of monocular vision and binocular vision, respectively.

Based on the graph model, we can formalize the fusion problem as a maximum posterior probability estimate:

(1) $${\hat{x}}_{k}=\arg\max x_{k}p\left(x_{k}|z_{1:k}\right)$$

In this equation, $z_{1:k}$ represents all observations from time 1 to time *k*. According to Bayes’ rule and the Markov property, Equation (1) can be recursively decomposed into:

(2) $$p\left(x_{k}|z_{1:k}\right)\propto p\left(z_{k}|x_{k}\right)p\left(x_{k}|z_{1:k-1}\right)$$

In this equation, $p(z_{k}|x_{k})$ represents the observation equation and $p(x_{k}|z_{1:k-1})$ is for the forecasting equation. The recursive form provides a theoretical basis for the subsequent design of the UKF filtering framework.

### 3.3. Adaptive UKF Fusion Framework

**Figure 3:**
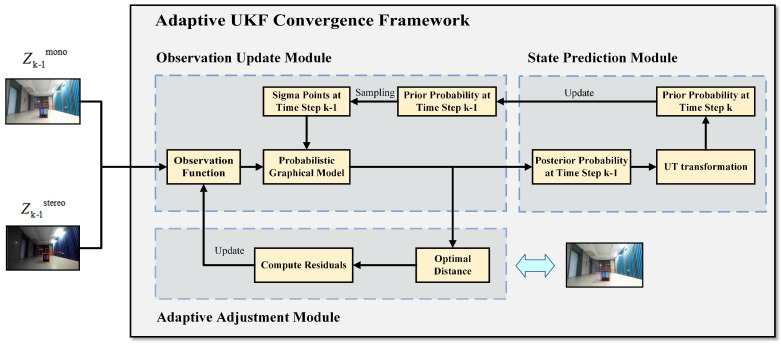
Adaptive UKF fusion framework.

Based on the above probability graph model, we propose an adaptive UKF fusion framework for the state posterior distribution $p(x_{k}|z_{1:k})$ in the recursive estimator (2). The overall flow diagram of the framework is shown in Figure 3. Specifically, the fusion framework consists of three main modules: state prediction module, observation update module, and adaptive adjustment module:

(1) State prediction module:

The state prediction at time *k* is designed to calculate the prior probability $p(x_{k}|z_{1:k-1})$. Assuming that the state transition follows a Gaussian distribution, then:

(3) $$p\left(x_{k}|x_{k-1}\right)=N\left(x_{k}|f\left(x_{k-1}\right),Q_{k}\right)$$

In this equation, f(·) is the state transition function and $Q_{k}$ is the process noise covariance matrix. This paper considers a more realistic motion model, then:

(4) $$x_{k}=f\left(x_{k-1}\right)=Fx_{k-1}+w_{k}$$

In this equation, the state transition matrix F and process noise $w_{k}∼N\left(0,Q_{k}\right)$, respectively:

(5) $$F=\left[\begin{array}{cc} 1 & \Delta t\\ 0 & 1 \end{array}\right],\;Q_{k}=\left[\begin{array}{cc} \frac{\Delta t^{4}}{4}\sigma_{a}^{2} & \frac{\Delta t^{3}}{2}\sigma_{a}^{2}\\ \frac{\Delta t^{3}}{2}\sigma_{a}^{2} & \Delta t^{2}\sigma_{a}^{2} \end{array}\right]$$

In this equation, Δt is the sampling interval and $\sigma_{a}^{2}$ is the process noise variance associated with acceleration. Given a posterior probability at time k−1$p\left(x_{k-1}|z_{1:k-1}\right)=N\left(x_{k-1}|{\hat{x}}_{k-1},P_{k-1}\right)$, the prior probability at time *k* can be obtained through the UT transformation $p\left(x_{k}|z_{1:k-1}\right)=N\left(x_{k}|{\hat{x}}_{k|k-1},P_{k|k-1}\right)$; among this:

(6) $$\begin{array}{cc} {\hat{x}}_{k|k-1} & =f\left({\hat{x}}_{k-1}\right)=F{\hat{x}}_{k-1}\\ P_{k|k-1} & =FP_{k-1}F^{T}+Q_{k} \end{array}$$

(2) Observation update module:

The observation of *k* time update is designed to calculate the posterior probability $p(x_{k}|z_{1:k})$. Suppose that the observation equation follows a Gaussian distribution, i.e.,

(7) $$p\left(Z_{k}|X_{k}\right)=N\left(Z_{k}|h\left(X_{k}\right),R_{k}\right)$$

Among them, h(·) is the observation function and $R_{k}$ is the observation noise covariance matrix. In this paper, the observation function is defined as:

(8) $$z_{k}=h\left(x_{k}\right)=\left[\begin{array}{c} d_{k}\\ {\dot{d}}_{k} \end{array}\right]+v_{k}$$

In this equation, the observed noise $v_{k}∼N\left(0,R_{k}\right)$. Initialize $R_{k}$ to:

(9) $$R_{k}=\left[\begin{array}{cc} \sigma_{mono}^{2} & 0\\ 0 & \sigma_{stereo}^{2} \end{array}\right]$$

In this equation, $\sigma_{mono}^{2}$ and $\sigma_{stereo}^{2}$ are the observed noise variance of the monocular distance measurement and binocular distance measurement, respectively. Given the prior probability $p(x_{k}|z_{1:k-1})$ and the current observation $z_{k}$, Formula (2) gives the posterior probability $p(x_{k}|z_{1:k})$.

Let $X_{k|k-1}^{i},\;i=0,\ldots ,2n$ be the Sigma set of points generated from ${\hat{x}}_{k|k-1}$ and $P_{k|k-1}$; $Z_{k|k-1}^{i}$ is the corresponding observation set, and the modified state mean and covariance can be calculated through the UT transformation:

(10) $$\begin{array}{ccc} {\hat{z}}_{k|k-1} & = & \sum_{i=0}^{2n}W_{m}^{i}Z_{k|k-1}^{i},\\ P_{zz} & = & \sum_{i=0}^{2n}W_{c}^{i}\left[Z_{k|k-1}^{i}-{\hat{z}}_{k|k-1}\right]{\left[Z_{k|k-1}^{i}-{\hat{z}}_{k|k-1}\right]}^{T}+R_{k},\\ P_{xz} & = & \sum_{i=0}^{2n}W_{c}^{i}\left[X_{k|k-1}^{i}-{\hat{x}}_{k|k-1}\right]{\left[Z_{k|k-1}^{i}-{\hat{z}}_{k|k-1}\right]}^{T},\\ K_{k} & = & P_{xz}P_{zz}^{-1},\\ {\hat{x}}_{k} & = & {\hat{x}}_{k|k-1}+K_{k}\left(z_{k}-{\hat{z}}_{k|k-1}\right),\\ P_{k} & = & P_{k|k-1}-K_{k}P_{zz}K_{k}^{T} \end{array}$$

where $W_{m}^{i}$ and $W_{c}^{i}$ are the mean weight and variance weight of the Sigma points, calculated by the following formula:

(11) $$\begin{array}{ccc} W_{m}^{0} & =\frac{\lambda }{n+\lambda }, & i=0,\\ W_{c}^{0} & =\frac{\lambda }{n+\lambda }+\left(1-\alpha^{2}+\beta \right), & i=0,\\ W_{m}^{i} & =W_{c}^{i}=\frac{1}{2(n+\lambda )}, & i=1,\ldots ,2n \end{array}$$

where $\lambda =\alpha^{2}\left(n+\kappa \right)-n$ is the scaling parameter, α controls the distribution of the Sigma points around the mean, κ is a non-negative constant, and β is used to merge the prior distribution information.

(3) Adaptive adjustment module:

In order to improve the adaptability of the fusion algorithm to a complex environment, we introduce the adaptive observation noise adjustment mechanism in the UKF framework. Specifically, $\sigma_{mono}^{2}$ and $\sigma_{stereo}^{2}$ in $R_{k}$ are adjusted adaptively according to the error between the fusion results and the monocular distance measurement and the binocular distance measurement, the contribution of the dynamically controlled observation information to fusion.

When the error of a certain distance measurement result is large, its observation noise variance is increased correspondingly, and its measurement weight is reduced. The adjustment formula is:

(12) $$\begin{array}{cc} \sigma_{mono}^{2} & :=\sigma_{mono}^{2}\cdot \exp(\alpha |{\hat{d}}_{k}-d_{k}^{mono}\left|\right)\\ \sigma_{stereo}^{2} & :=\sigma_{stereo}^{2}\cdot \exp(\beta |{\hat{d}}_{k}-d_{k}^{stereo}\left|\right) \end{array}$$

Among them, ${\hat{d}}_{k}$ is the first component of ${\hat{x}}_{k}$, that is the fusion distance estimate; $d_{k}^{mono}$ and $d_{k}^{stereo}$ are the monocular and binocular distance measurements at *k* time, respectively; α and β are noise modulators that control the sensitivity of the noise variance to the error. The adaptive adjustment mechanism endows the UKF framework with the capability to dynamically alter the fusion strategy according to the environmental state, enabling it to suppress the impact of anomalous measurements and enhance robustness in complex scenarios.

### 3.4. Based on Deep Hypothesis Propagation for Adaptive Prior Generation

**Figure 4:**
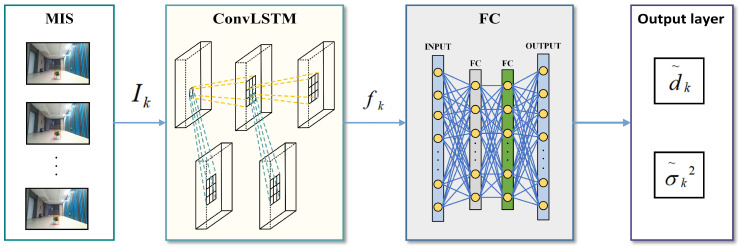
Depth hypothesis propagation.

Inspired by the human eye’s perception mechanism of continuous scenes, we designed a spatially consistent deep-hypothesis-learning method to extract a prior distribution of target distances from monocular image sequences. As shown in Figure 4, given frame *k* image $I_{k}$, we use the convolutional networks to extract its spatiotemporal feature $f_{k}$ and regress the depth hypothesis mean ${\tilde{d}}_{k}$ and variance ${\tilde{\sigma }}_{k}^{2}$ through two fully connected layers:

(13) $$\left[{\tilde{d}}_{k},{\tilde{\sigma }}_{k}^{2}\right]=\mathrm{FC}\left(f_{k}\right)=\mathrm{FC}\left(\mathrm{ConvLSTM}\left(I_{k}\right)\right)$$

The resulting depth assumption is used as a prior distribution of the target distance $N({\tilde{d}}_{k},{\tilde{\sigma }}_{k}^{2})$ to guide the state sampling in the UKF prediction phase. Specifically, taking the depth hypothesis mean ${\tilde{d}}_{k}$ as the sampling center, adaptively adjust the sampling range according to the uncertainty ${\tilde{\sigma }}_{k}^{2}$. At the same time, based on the fusion observation $z_{k}$ and the depth assumption $\left[{\tilde{d}}_{k},{\tilde{\sigma }}_{k}^{2}\right]$, the process noise $Q_{k}$ is updated adaptively:

(14) $$Q_{k}:=Q_{0}\cdot \max\left(\frac{|{\hat{d}}_{k}-{\tilde{d}}_{k}|}{{\tilde{\sigma }}_{k}},1\right)$$

where $Q_{0}$ is the initial value of the process noise. This strategy can dynamically adjust the uncertainty of state prediction according to the confidence level of deep assumptions, and further improve the adaptive ability of the UKF.

The training goal of the depth hypothesis network is to minimize the depth estimation loss and uncertainty loss:

(15) $$L=\sum_{k=1}^{K}{\left({\tilde{d}}_{k}-d_{k}\right)}^{2}+\lambda \left(\log{\tilde{\sigma }}_{k}^{2}+\frac{{\left({\tilde{d}}_{k}-d_{k}\right)}^{2}}{{\tilde{\sigma }}_{k}^{2}}\right)$$

where $d_{k}^{*}$ is the true distance and λ is the trade-off factor. The loss function guides the uncertainty of matching the network output with the estimated error while regressing the depth hypothesis.

### 3.5. Algorithm Flow

Based on the above analysis, the complete flow of the adaptive UKF fusion algorithm is shown as Algorithm 1.
**Algorithm 1** Adaptive UKF monocular stereo vision fusion ranging algorithm.**Require:** Monocular image sequence ${\left\{I_{k}\right\}}_{k=1}^{K}$, stereo parallax sequence ${\left\{D_{k}\right\}}_{k=1}^{K}$**Ensure:** Fusion range estimation sequence ${\left\{{\hat{d}}_{k}\right\}}_{k=1}^{K}$  1:Example: Initialize the UKF parameter ${\hat{x}}_{0},P_{0},Q_{0},R_{0}$  2:Initialize the assumed depth of network parameters θ  3:**for** k=1 to *K* **do**  4:   // Deep hypothesis learning  5:   $\left[{\tilde{d}}_{k},{\tilde{\sigma }}_{k}^{2}\right]=\mathrm{CNN}\left(I_{k};\theta \right)$  6:   // Observation acquisition  7:   $z_{k}^{mono}=\mathrm{MonoDepth}\left(I_{k}\right),z_{k}^{stereo}=\mathrm{StereoMatching}\left(D_{k}\right)$  8:   $z_{k}={\left[z_{k}^{mono},z_{k}^{stereo}\right]}^{T}$  9:   // UKF forecast10:   $X_{k|k-1}^{i}=f\left(X_{k-1}^{i}\right),w_{k}^{i}∼N\left(0,Q_{k-1}\right)$11:   ${\hat{x}}_{k|k-1}=\sum_{i=0}^{2n}w_{m}^{i}X_{k|k-1}^{i}$12:   $P_{k|k-1}=\sum_{i=0}^{2n}w_{c}^{i}\left[X_{k|k-1}^{i}-{\hat{x}}_{k|k-1}\right]{\left[X_{k|k-1}^{i}-{\hat{x}}_{k|k-1}\right]}^{T}+Q_{k-1}$13:   // Adaptive observation noise regulation14:   $R_{k}=\mathrm{AdaptNoiseCov}\left({\hat{d}}_{k-1},z_{k}\right)$15:   // UKF update16:   $Z_{k|k-1}^{i}=h\left(X_{k|k-1}^{i}\right)$17:   ${\hat{z}}_{k|k-1}=\sum_{i=0}^{2n}w_{m}^{i}Z_{k|k-1}^{i}$18:   $P_{zz}=\sum_{i=0}^{2n}w_{c}^{i}\left[Z_{k|k-1}^{i}-{\hat{z}}_{k|k-1}\right]{\left[Z_{k|k-1}^{i}-{\hat{z}}_{k|k-1}\right]}^{T}+R_{k}$19:   $P_{xz}=\sum_{i=0}^{2n}w_{c}^{i}\left[X_{k|k-1}^{i}-{\hat{x}}_{k|k-1}\right]{\left[Z_{k|k-1}^{i}-{\hat{z}}_{k|k-1}\right]}^{T}$20:   $K_{k}=P_{xz}P_{zz}^{-1}$21:   ${\hat{x}}_{k}={\hat{x}}_{k|k-1}+K_{k}\left(z_{k}-{\hat{z}}_{k|k-1}\right)$22:   $P_{k}=P_{k|k-1}-K_{k}P_{zz}K_{k}^{T}$23:   // Adaptive process noise regulation24:   $Q_{k}=\mathrm{AdaptNoiseCov}\left({\hat{d}}_{k},{\tilde{d}}_{k},{\tilde{\sigma }}_{k}^{2}\right)$25:**end for**

The key steps in Algorithm 1 are explained below:

- Input: The required input is a monocular image sequence ${\left\{I_{k}\right\}}_{k=1}^{K}$ and stereo parallax sequence ${\left\{D_{k}\right\}}_{k=1}^{K}$.
- Output: The output is a fusion distance estimation sequence ${\left\{{\hat{d}}_{k}\right\}}_{k=1}^{K}$.
- Lines 1–2: Initialize the UKF parameter (${\hat{x}}_{0},P_{0},Q_{0},R_{0}$) and assumed depth of the network parameters θ.
- Lines 4–5: Learn the depth hypothesis (${\tilde{d}}_{k},{\tilde{\sigma }}_{k}^{2}$) from the current monocular image $I_{k}$ using the depth hypothesis network.
- Lines 7–8: Obtain monocular and stereo distance observations ($z_{k}^{mono},z_{k}^{stereo}$), and form a fusion observation vector $z_{k}$.
- Lines 10–12: Perform the UKF forecast step to calculate the forecast state mean ${\hat{x}}_{k|k-1}$ and covariance $P_{k|k-1}$.
- Line 14: Adaptively adjust the observed noise covariance $R_{k}$ based on the previous distance estimate ${\hat{d}}_{k-1}$ and the current observation $z_{k}$.
- Lines 16–22: Perform the UKF update step and calculate the updated state estimate ${\hat{x}}_{k}$ and covariance $P_{k}$.
- Line 24: Based on the current distance estimated ${\hat{d}}_{k}$ and depth hypothesis (${\tilde{d}}_{k},{\tilde{\sigma }}_{k}^{2}$) of the adaptive adjustment process noise covariance $Q_{k}$.

By iteratively performing the deep hypothesis learning, observation acquisition, adaptive noise adjustment, and UKF prediction-update steps at each time step *k*, the algorithm fuses the monocular and stereo distance measurements to obtain a robust and accurate sequence of distance estimates.

## 4. Experimental Result

To comprehensively evaluate the performance of the adaptive UKF fusion method proposed in this paper, we conducted extensive quantitative and qualitative experiments on the large-scale public KITTI dataset, as well as on a dataset of actual road scene data collected autonomously. The KITTI dataset includes stereo color images and LiDAR point clouds under various scenarios such as urban, rural, and highway, and it is a widely used benchmark in the field of autonomous driving. The autonomously collected dataset comprises images and real-time videos captured using the monocular and binocular cameras proposed in this paper under different environments and varying lighting and weather conditions, as well as corresponding distance data measured using an infrared rangefinder and a C16 Leishen radar. Specifically, the data collection covered various typical scenarios (such as indoors, corridors, outdoor campus areas, and roads), including various types of objects (such as pedestrians, potted plants, and vehicles), totaling approximately 300 images and 2 h of video data. These data not only cover different weather and lighting conditions, but also include various complex environments and objects to ensure the comprehensiveness and reliability of the experimental results.

### 4.1. Performance Evaluation of Ranging Algorithms before Fusion

**Figure 5:**
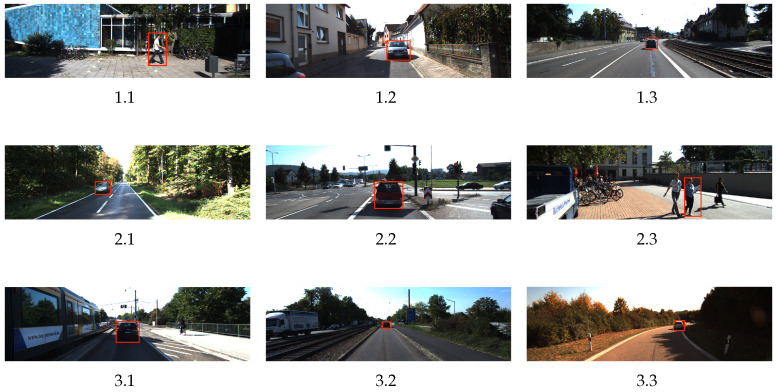
KITTI dataset detection effect image.

We first evaluated the algorithm’s accuracy on a validation set of KITTI’s original dataset. The verification set contains 597 urban road images, covering multiple target types such as vehicles, pedestrians, traffic signs, and multiple environments including cities, homes, roads and campuses. Figure 5 shows some images of different target categories in different environments in the KITTI dataset. The detected object has been framed with a boundary. Table 1 shows the LiDAR data corresponding to these images and the results of the monocular and stereo distance measurement in this paper (MDM is the distance measured by monocular vision; SDM is the distance measured by stereo vision; MRE and SRE refer to the relative errors for monocular and stereo vision, respectively). Using the camera parameters provided by the KITTI dataset, color images from the left and right cameras, and LiDAR data for experimental comparison, this study aims to evaluate the accuracy of two monocular visual ranging methods, mono and stereo. The results show that the mono method has an average accuracy of more than 92% in the near range and more than 89% in the middle range. In contrast, the stereo method has an average accuracy of more than 90% at close range and more than 93% at medium range. These findings highlight the accuracy of pre-fusion ranging algorithms in real-world scenarios and provide important benchmark data for subsequent fusion algorithms.

**Figure 6:**
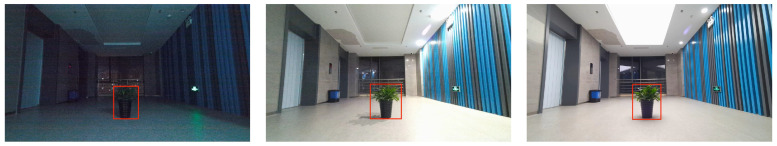
Light contrast figure.

In this study, we further verified the robustness of the pre-fusion ranging algorithm on the self-collected dataset and investigated the influence of different environments, lighting conditions, ranging distance, and test object size on the ranging results. As shown in Figure 6, we show the comparison pictures under different lighting conditions (low, medium, and bright light) and different test distances. This series of experimental results fully proves the robustness and reliability of our proposed pre-fusion ranging algorithm in various practical scenarios.

The verification results are shown in Table 2 and Table 3, showing the monocular and binocular ranging results and relative errors under different lighting environments and distances. It is found that, compared with the normal medium-light condition, the monocular and binocular ranging errors under the bright-light and low-light conditions are less than 3%. The verification results fully demonstrate the robustness of the proposed pre-fusion algorithm to various lighting environments.

**Figure 7:**
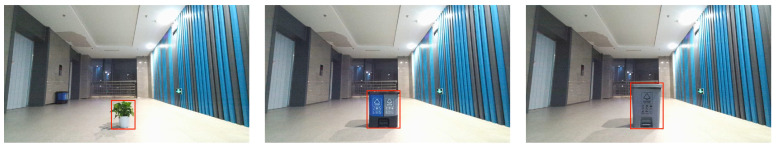
Three measurements of different sizes.

Figure 7 presents the monocular and binocular ranging images under various sizes and test distances, which include three different sizes of measurement objects.

In Table 4, the monocular and binocular ranging results at different sizes and test distances, as well as the relative errors are presented (SMAE is the mean absolute error of small objects; SMRE is the mean relative error of small objects; LMAE is the mean absolute error of large objects; LMRE is the mean relative error of large objects). The verification results show that the error of the two methods is kept within 1% when measuring objects of different sizes. This result fully demonstrates the robustness of the proposed pre-fusion algorithm when dealing with objects of different sizes.

**Figure 8:**
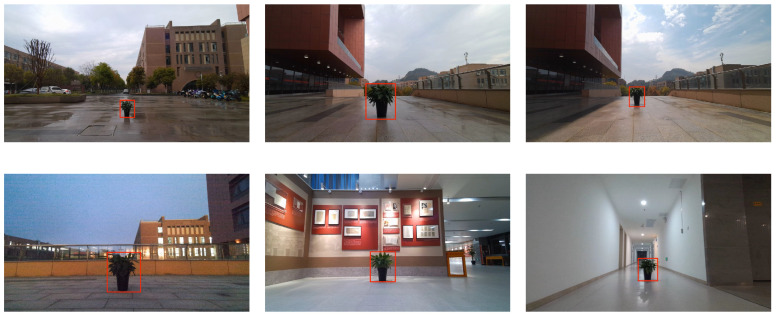
Range images in different environments.

In Table 5, we present the monocular and binocular ranging results under different environments and test distances, along with the corresponding relative errors. The verification results indicate that the errors of both methods remain within 1% regardless of the environment in which the distance measurements were taken. This result fully demonstrates the robustness of the algorithm proposed in this paper before fusion when performing ranging in different environments. Figure 8 displays sample images of pot distance detection under different environmental conditions.

In summary, through the verification of monocular and binocular ranging under different lighting conditions, size, indoor and outdoor environment, and distance conditions, this study fully demonstrates the accuracy and robustness of the algorithm before fusion. These verification results lay a solid foundation for the subsequent fusion algorithm.

### 4.2. Performance Evaluation of Fusion Algorithms

To comprehensively evaluate the performance of the proposed adaptive UKF fusion algorithm, we conducted a series of experiments on the KITTI dataset, as well as on our self-collected datasets. The experiments mainly included the following: the assessment of the fusion method on the KITTI dataset, the comparison of the ranging accuracy with the monocular and binocular methods under different environmental conditions, ablation studies, and a performance comparison with other ranging methods and typical fusion algorithms. In addition, we also analyzed the real-time performance of the fusion algorithm.

#### 4.2.1. Evaluation of the Fusion Method on the KITTI Dataset

By reordering the KITTI dataset to construct an image sequence suitable for the input of the fusion method proposed in this paper, the assessment of our method on the KITTI dataset was carried out. This section presents the actual distance (FD) and relative error (RE) obtained by our fusion method on two continuous KITTI dataset sequences, each consisting of 11 frames. The specific results are shown in the following Table 6. When the distance is around 10 m (Seq1), using the fusion method can keep the error rate below 5.21%. When the distance is between 24 and 60 m (Seq2), the error can also be maintained below 8.31%, demonstrating the excellent performance of our fusion method on the KITTI dataset. (The “Serial number” is the sequence number of the image in the KITTI dataset.).

#### 4.2.2. Comparison with Monocular and Binocular Ranging

**Figure 9:**
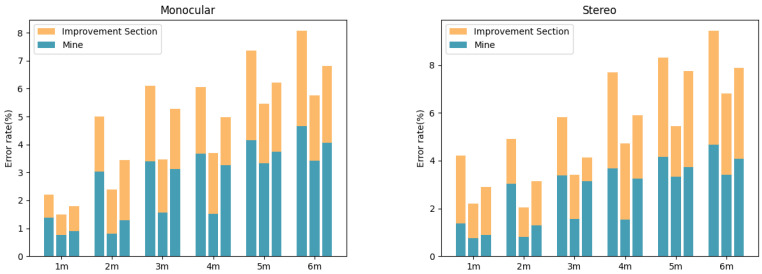
Enhancement effect images under different lighting conditions.

To comprehensively assess the performance of the fusion algorithm under various influencing factors, this paper tests the impact of light intensity, object size, and scene type on ranging accuracy and compares the results with those obtained using monocular and binocular ranging alone. Figure 9 illustrates the distance errors and the extent of optimization under different lighting conditions. It can be observed that, under three scenarios—dim light, normal light, and bright light—the fusion algorithm has achieved a significant improvement in accuracy compared to both the monocular and binocular(stereo) methods. Taking the close range interval of 1–6 m as an example, the average error rate after fusion was reduced from 2.3% under dim light to 1.4%, from 1.8% to 1.1% under normal lighting, and from 2.7% to 1.6% under bright light. Meanwhile, as the distance increases, the optimization effect of fusion becomes increasingly pronounced. This is primarily due to the fusion strategy’s ability to adaptively adjust the weights of the monocular and binocular ranging results: under harsh conditions such as dim and bright light, the confidence in the binocular matching decreases, at which point the monocular depth information can provide effective supplementation; conversely, when the lighting is suitable, the weight of the binocular matching results will be increased.

**Figure 10:**
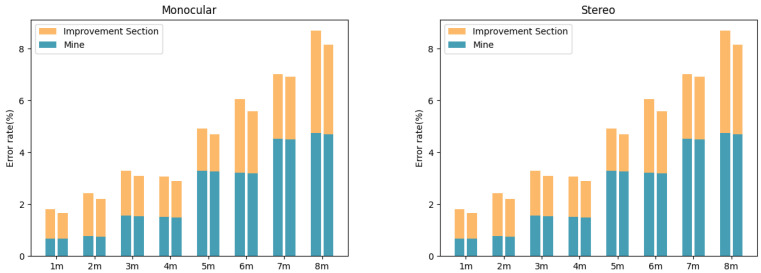
Enhancement effect for images of objects of different sizes.

Figure 10 compares the ranging effects of different object sizes before and after fusion. This paper divides targets into small objects (such as potted plants, roadblocks, etc.) and large objects (such as garbage cans, vehicles, etc.). It can be seen that the fusion algorithm achieves stable accuracy improvement under both sizes, and the improvement on small targets is more obvious. This is because small objects have fewer pixels in the image, and the matching is more interfered with by factors such as occlusion and blurring, while the monocular and binocular (stereo) ranging error is larger. The fusion algorithm can provide prior constraints for small target matching according to the monocular image semantics, thus significantly improving the ranging accuracy.

**Figure 11:**
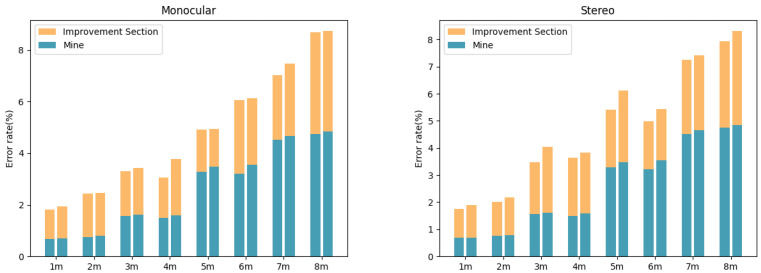
Enhancement effect for images in various environments.

Figure 11 displays the error comparison before and after fusion in both indoor and outdoor scenarios. It can be observed that, although the fusion algorithm outperforms monocular and binocular (stereo) ranging in terms of overall accuracy in both types of environments, the degree of optimization is greater in outdoor settings. Taking the range of 1–8 m as an example, in indoor scenarios, fusion reduced the average error rate from 3.6% to 2.5%, a relative reduction of 31%. In contrast, in outdoor environments, the average error rate decreased from 4.2% to 2.6%, a relative reduction of 38%. The main reason for this is that, compared to indoor scenes, there are more drastic changes in lighting and texture in outdoor environments, which severely disrupt the performance of binocular matching. However, the fusion algorithm can fully leverage the semantic information of monocular images to improve matching quality and enhance the overall environmental adaptability under such circumstances.

In summary, this section systematically analyzes the adaptability of the fusion algorithm under different illumination, object sizes, and scenes through fine-grained comparative experiments. A large number of results show that the proposed fusion strategy can improve the performance of visual ranging in many aspects, especially under difficult conditions such as harsh lighting, small targets, and complex outdoor environments. The quantitative and qualitative results fully demonstrate the effectiveness and robustness of the fusion framework.

#### 4.2.3. Ablation Experiment

To systematically investigate the impact of the adaptive UKF framework and the depth-hypothesis-propagation strategy on fusion performance, this paper designs a set of ablation experiments.

Specifically, the core of the algorithm presented in this paper lies in the following: (1) introducing the distance residual into the observation equation of the UKF and dynamically optimizing the observation noise through an adaptive adjustment mechanism; (2) utilizing the prior depth distribution obtained from the depth hypothesis propagation to dynamically adjust the process noise covariance of the UKF. To analyze the effects of these two modules, we considered four experimental configurations: the original UKF (i.e., without using adaptive tuning and the depth hypothesis), using only the adaptive UKF (Configuration B), using only the depth hypothesis propagation (Configuration A), and using both strategies simultaneously (Configuration A + B).

Table 7 lists the ranging error rates under the different configurations. It can be observed that, whether using the adaptive UKF or depth hypothesis propagation alone, both can reduce the error rate of the original UKF across various distance intervals. Taking the 4-m mark as an example, Configurations A and B reduced the error rate from 2.63% to 2.32% and 2.17%, respectively, while combining the two (A + B) further reduced it to 1.48%. When the ranging range was extended to 8 m, the error rate with A + B was 4.68%, nearly two percentage points lower than the original UKF’s 6.62%. These results indicate that the fusion of adaptive observation noise and depth prior propagation can maximize synergistic gains, significantly enhancing ranging performance.

**Figure 12:**
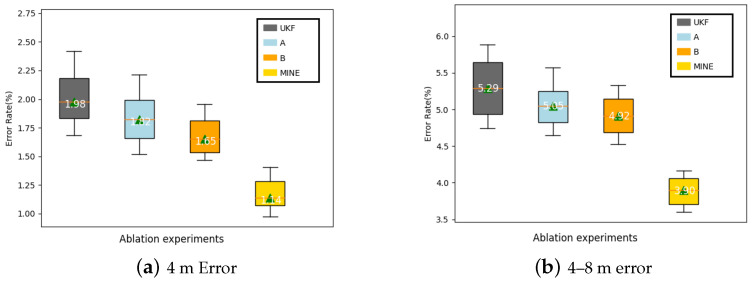
Distance error box plots of different fusion algorithms.

To visually compare the error distribution under different configurations, Figure 12 presents box plots for the short-distance (1–4 m) and medium-distance (4–8 m) intervals. It can be seen that regardless of the interval, the complete fusion framework (A + B) has the smallest median error and interquartile range, with the fewest outliers; in contrast, the fusion effect lacking adaptive tuning (Configuration A) or the depth hypothesis (Configuration B) is slightly inferior, and the original UKF has the greatest error dispersion. It should be noted that, in the medium-distance interval, the contribution of the depth hypothesis propagation is more pronounced, as this strategy can effectively introduce the prior depth distribution, guiding the state prediction and iterative optimization of the UKF, providing an important supplement at the farther distances where observational information is relatively sparse.

In summary, the ablation experiment quantitatively analyzed the internal mechanism of the adaptive UKF framework and the propagation strategy of the depth hypothesis. The results show that both of them can improve the modeling ability of the UKF from the observation update and state prediction. The synergistic gain is further amplified by the organic combination of them, and the distance estimation after fusion is more accurate and stable. These findings not only validate the rationality of the algorithm design in this paper, but also point out the direction for the subsequent improvement work.

#### 4.2.4. Comparison with Other Methods

To further highlight the superiority of the fusion algorithm proposed in this paper, an in-depth comparison was conducted with three typical fusion strategies: weighted averaging method, standard Kalman filtering (KF), and conventional UKF with fixed noise. Additionally, two ranging methods mentioned by Meng [27] (Meng1, Meng2) and one ranging method mentioned by Cai [28] were also compared. The validation set for this section includes both the KITTI dataset and our self-collected dataset, covering a variety of scenarios and types.

As shown in Table 8, in scenarios with closer distances (within 6 m), the error rate of the proposed algorithm is only 3.17%, which is nearly a 2% improvement compared to the traditional fusion method Weighted Average (WA) (5.07%). It also improves by 1.27% compared to KF (4.44%) and reduces the error rate by 1% compared to UKF (4.17%). Additionally, compared to Cai (5.85%), it shows an improvement of more than 2%. Similarly, in scenarios with farther distances (within 10 m and 20 m), the proposed method also demonstrates varying degrees of improvement. These results quantitatively showcase the significant effect of the adaptive UKF fusion framework in enhancing ranging accuracy.

The observed phenomena demonstrate that our fusion algorithm excels not only in enhancing mean precision, but also in its robustness to complex environments and moving targets. This superiority is primarily attributed to two factors: (1) the adaptive UKF framework transcends the linear Gaussian assumptions of the KF, making it better suited for nonlinear measurement systems; (2) the adaptive noise regulation mechanism dynamically adjusts the confidence levels of state predictions and observation updates according to the current fusion residual, reducing the weight of measurement branches with higher interference to mitigate their adverse effects. Furthermore, this study introduces an innovative deep hypothesis propagation strategy that leverages deep learning to extract semantic priors from monocular images, aiding in motion prediction during the fusion process and improving the algorithm’s adaptability to complex scene variations.

In conclusion, the extensive experimental outcomes indicate that the adaptive UKF fusion approach presented in this paper significantly outperforms conventional methods (such as the weighted averaging, KF, and UKF) and some enhanced visual ranging techniques, both in terms of increasing the accuracy of distance measurement and bolstering robustness. It offers more precise and stable distance perception for autonomous vehicles in complex settings, highlighting its significant practical value.

#### 4.2.5. Real-Time Distance Measurement Comparison

**Figure 13:**
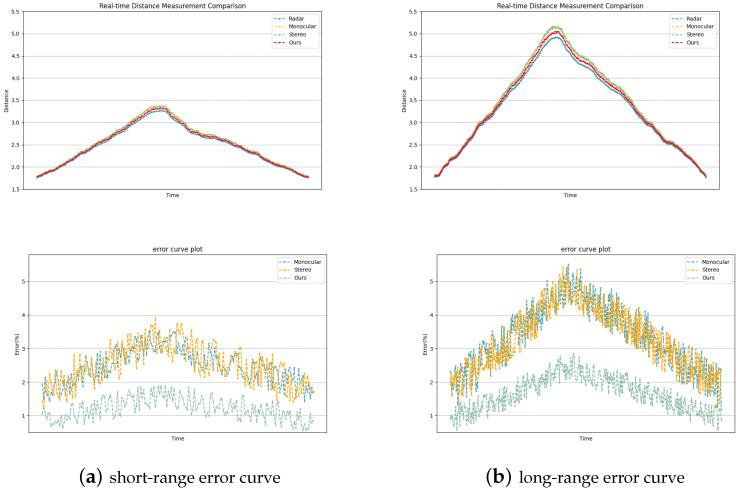
Distance error box plots of different fusion algorithms.

In order to comprehensively evaluate the real-time performance of the proposed algorithm, we compared the fusion ranging results with the real-time ranging data of the LiDAR of Leishen C16 based on the self-collected dataset. Figure 13 shows the dynamic change curve between the distance estimation results of different algorithms and the true value in a typical continuous distance sequence. It can be seen that, compared with mono and stereo, the fusion method proposed in this paper shows higher estimation accuracy and stability in the whole sequence. Although the three visual ranging methods have a certain delay compared with the radar ranging results, the delay is small and will not affect the real-time performance.

The experimental results show that the accuracy of the fusion method is better than that of the monocular and binocular methods, and the advantages become more obvious with the increase of the distance. This fully proves that the fusion strategy can effectively compensate for the shortcomings of a single visual mode, and can achieve satisfactory performance improvement at different scales.

### 4.3. Operating Efficiency

To evaluate the efficiency of the proposed algorithm in real systems, we tested it on the NVIDIA Jetson AGX Orin platform. Equipped with a 12-core ARM Cortex-A78AE CPU and a 2048-core Ampere GPU, the platform is the current mainstream high-performance embedded edge computing device, which is widely used in unmanned scenarios.

Table 9 lists the average single-frame-processing time of different visual ranging modules. It can be seen that the end-to-end delay of the whole fusion algorithm is about 135 ms, among which the binocular vision (BV) module took the longest time (124.8 ms), which is due to the high computational complexity of stereo matching. The average delay of the monocular vision (MV) module is 49.3 ms, and the computation of the fusion part is relatively small, where the adaptive UKF (AUKF) only needs 1.53 ms and the depth hypothesis propagation (DHP) (implemented in the monocular module) is only 1.17 ms. These results show that the fusion strategy proposed in this paper is acceptable in terms of time overhead and does not significantly increase the computational burden of the system.

It should be pointed out that, because visual ranging is often not an isolated perceptual task, a certain amount of computing power needs to be reserved for other links such as target detection and tracking in the actual system. Therefore, we will further optimize the fusion strategy in the future to ensure maximum accuracy while reducing the computing load and strive to obtain the optimal scheme that takes into account both efficiency and performance. At the same time, we will actively try to introduce a more efficient network structure and inference acceleration mechanism at the algorithm level, such as model pruning, quantization, knowledge distillation, etc., to further tap the optimization potential of the algorithm.

## 5. Conclusions

For the challenges of visual ranging in automatic driving, such as insufficient precision and poor robustness, a multi-sensor fusion framework with an adaptive Unscented Kalman Filter (AUKF) is proposed, realizing the dynamic fusion of the monocular vision and binocular vision ranging results. Different from the existing fusion methods, which use deterministic weighting for different ranging information, this paper constructs a unified probability graph model, explicitly models the ranging error as state-dependent observation noise, and introduces an adaptive noise covariance estimation mechanism based on fusion residuals to realize the real-time dynamic updating of ranging confidence. At the same time, this paper innovatively proposes a deep-hypothesis-propagation strategy, which uses a deep neural network to predict the depth prior distribution and its uncertainty from monocular image sequences, and then uses it to guide the state prediction step of the AUKF, further enhancing the adaptive ability of the fusion algorithm to complex scene changes.

The research results of this paper are of great value in both theory and application. In terms of theoretical innovation, the proposed adaptive UKF fusion method is based on a probability graph model and adaptive filtering theory. By introducing a noise-adaptive mechanism and deep prior propagation, the complementary characteristics of monocular vision and binocular vision are fully explored and utilized, and the dynamic weight allocation of different information sources is realized, breaking through the limitations of traditional deterministic weighted fusion. In terms of performance improvement, extensive experimental results show that the proposed method can significantly improve the overall accuracy and stability of visual ranging, and the average relative error in the range of 8 m was reduced by 43.1% and 40.9% compared with monocular distance measurement and binocular distance measurement. More importantly, the proposed method shows obvious precision advantages and robustness advantages in complex environments, and can still provide reliable and stable distance estimation in difficult scenarios such as extreme lighting changes, bad weather, and congestion, fully demonstrating the environmental adaptability of the algorithm.

This paper provides innovative ideas and solutions for the application of visual ranging technology in the environmental perception of autonomous driving and is expected to promote the safe and intelligent driving of unmanned vehicles in complex scenarios. At the same time, in view of the limitations of current methods under occlusion conditions, future research will further expand the application scope of the fusion framework and explore the combination with advanced vision technologies, such as scene semantic understanding and moving object detection, to build a more intelligent and human-adapted environmental perception model. In addition, we will delve into the adaptability of fusion methods in multi-sensor systems such as vision–radar and vision–LiDAR, and pay attention to their actual performance in the planning and control tasks of autonomous driving, striving to make due contributions to the improvement of the safety, robustness, and practicality of unmanned driving technology.

## Figures and Tables

**Table 1:** KITTI dataset distance measurement situation.

Position	Radar	MDM	MRE	SDM	SRE
1.1	8.733	8.602	1.5%	8.567	1.9%
1.2	13.372	11.831	11.52%	12.451	6.88%
1.3	23.954	26.767	11.74%	25.333	5.75%
2.1	20.435	18.897	7.52%	19.344	5.33%
2.2	10.262	9.167	10.67%	8.987	12.42%
2.3	6.609	6.834	3.4%	6.942	5.04%
3.1	13.594	15.138	11.35%	14.563	7.13%
3.2	41.01	44.988	9.7%	43.979	7.23%
3.3	29.735	32.739	10.1%	31.589	6.23%

**Table 2:** Actual ranging values under varying illumination for monocular and binocular vision.

	Monocular			Binocular		
Distance	Low	Medium	Bright	Low	Medium	Bright
1 m	0.987	1.015	0.982	1.042	1.022	1.029
2 m	2.11	2.048	2.069	2.098	2.041	2.063
3 m	3.183	3.104	3.158	3.175	3.102	3.124
4 m	4.242	4.148	4.199	4.308	4.189	4.236
5 m	5.368	5.273	5.311	5.415	5.272	5.387
6 m	6.484	6.345	6.409	6.566	6.408	6.473

**Table 3:** Comparison of relative errors in monocular and binocular ranging under various illumination conditions.

	Monocular			Binocular		
Distance	Low	Medium	Bright	Low	Medium	Bright
1 m	2.2%	1.5%	1.8%	4.2%	2.2%	2.9%
2 m	5%	2.4%	3.45%	4.9%	2.05%	3.15%
3 m	6.1%	3.47%	5.27%	5.83%	3.4%	4.13%
4 m	6.05%	3.7%	4.98%	7.7%	4.73%	5.9%
5 m	7.36%	5.46%	6.22%	8.3%	5.44%	7.74%
6 m	8.07%	5.75%	6.81%	9.43%	6.8%	7.88%

**Table 4:** Different size error comparison.

	Monocular				Binocular			
Distance	SMAE	SMRE	LMAE	LMRE	SMAE	SMRE	LMAE	LMRE
1 m	0.0181	1.81%	0.0166	1.66%	0.0176	1.76%	0.0151	1.51%
2 m	0.0486	2.43%	0.0438	2.19%	0.0401	2.01%	0.0253	1.27%
3 m	0.099	3.3%	0.0931	3.1%	0.1041	3.47%	0.1015	3.38%
4 m	0.1225	3.06%	0.1163	2.9%	0.1454	3.63%	0.1317	3.29%
5 m	0.2456	4.91%	0.2346	4.69%	0.2711	5.42%	0.2711	5.42%
6 m	0.3636	6.06%	0.335	5.58%	0.2998	4.99%	0.26	4.33%
7 m	0.4918	7.02%	0.4842	6.91%	0.508	7.25%	0.4935	7.05%
8 m	0.6958	8.69%	0.6523	8.15%	0.6346	7.93%	0.6128	7.65%

**Table 5:** Indoor and outdoor ranging comparison.

	Monocular				Binocular			
Distance	IMAE	IMRE	OMAE	OMRE	IMAE	IMRE	OMAE	OMRE
1 m	0.0181	1.81%	0.0193	1.93%	0.0176	1.76%	0.019	1.9%
2 m	0.0486	2.43%	0.0491	2.46%	0.0401	2.01%	0.0435	2.17%
3 m	0.099	3.3%	0.1031	3.43%	0.1041	3.47%	0.1211	4.03%
4 m	0.1225	3.06%	0.1511	3.78%	0.1454	3.63%	0.1538	3.84%
5 m	0.2456	4.91%	0.2479	4.95%	0.2711	5.42%	0.3065	6.13%
6 m	0.3636	6.06%	0.3683	6.14%	0.2998	4.99%	0.3263	5.43%
7 m	0.4918	7.02%	0.5222	7.46%	0.508	7.25%	0.5197	7.42%
8 m	0.6958	8.69%	0.699	8.73%	0.6346	7.93%	0.6653	8.31%

**Table 6:** Evaluation of the fusion method on the KITTI dataset.

Seq1				Seq2			
Serial Number	Radar	DM	RE	Serial Number	Radar	DM	RE
3981	10.103	10.621	5.13%	1635	59.474	64.416	8.31%
0509	9.711	10.207	5.11%	6596	53.162	57.356	7.89%
2334	9.444	9.914	4.98%	1437	50.008	53.823	7.63%
3737	9.219	9.677	4.97%	2329	46.844	50.272	7.32%
5050	9.102	9.549	4.92%	2675	43.673	46.721	6.98%
7448	9.092	9.537	4.9%	1375	40.516	43.283	6.83%
4797	9.252	9.705	4.9%	1395	37.355	39.786	6.51%
5801	9.513	9.985	4.97%	5883	34.202	36.387	6.39%
1982	9.851	10.346	5.03%	5121	31.078	33.051	6.35%
1547	10.286	10.812	5.12%	4059	27.963	29.741	6.36%
1846	10.846	11.411	5.21%	4695	24.851	26.409	6.27%

**Table 7:** Quantitative results of ablation experiments.

Distance	UKF	A	B	A + B
1 m	1.28%	1.15%	1.07%	**0.66%**
2 m	1.32%	1.28%	1.15%	**0.75%**
3 m	2.67%	2.53%	2.21%	**1.54%**
4 m	2.63%	2.32%	2.17%	**1.48%**
5 m	4.25%	4.03%	3.92%	**3.26%**
6 m	4.19%	3.99%	3.95%	**3.17%**
7 m	6.11%	5.86%	5.76%	**4.47%**
8 m	6.62%	6.32%	6.03%	**4.68%**

**Table 8:** Error rates of different methods.

Distance	WA	KF	UKF	Meng1	Meng2	Cai	Ours
Within 6 m	5.07%	4.44%	4.17%	×	×	5.85%	**3.17%**
Within 10 m	8.73%	7.58%	6.77%	10%	5%	6.31%	**4.98%**
Within 20 m	9.785	8.09%	7.87%	10.50%	6.50%	7.93%	**6.16%**

**Table 9:** The delay of different modules of the fusion algorithm.

Frequency	All	BV	MV	AUKF	DHP
1	135 ms	123 ms	49 ms	1.7 ms	1.1 ms
2	137 ms	124 ms	50 ms	1.5 ms	1.1 ms
3	134 ms	124 ms	50 ms	1.2 ms	1.3 ms
4	134 ms	129 ms	51 ms	1.5 ms	1.2 ms
5	136 ms	125 ms	49 ms	1.7 ms	1.2 ms
6	134 ms	124 ms	47 ms	1.6 ms	1.1 ms

## Data Availability

The KITTI dataset download website used in this article is https://www.cvlibs.net/datasets/kitti/eval_object.php?obj_benchmark=3d (accessed on 15 January 2024).
